# Construction Notes

**Published:** 1895-06-01

**Authors:** 


### CONSTRUCTION NOTES.

### Infectious Diseases Hospital, Alloa.

This building, which is attractive in appearance, has been erected from plans prepared by Messrs John Melvin and Son, architects, of Alloa. The administrative block is placed in the centre, from which the wards are reached by means of paved footways. The accommodation provided in this block consists of a doctor's room, matron's room, kitchen, scullery, larder, store room, and coal house on the ground floor, while on the top floor there are three bed-rooms for matron, nurses, and servant, bath-room, lavatories, &c. To the left of the main doorway is the doctor's room, which is arranged to be used as a waiting room if required. The male and female wards for doubtful cases are situated on either side of the administrative block, entirely isolated from it, having outside entrances, but overlooked by means of airtight windows from the doctor's and matron's room. Each of these wards is eighteen by fourteen feet. Two beds are provided in each, and attached to these rooms there is provided a bath-room, with lavatory, sink, &c. The heating is by means of open fire-places, and the construction and ventilation is similar to the main wards. The two pavilions are divided into four wards for males and females, and there are six beds in each. The wards are entirely isolated from each other, having the nurses' rooms between, and separate entrances to outside open porches. Each ward is provided with a bath-room. In the separation building, provision has been made for an ambulance waggon, a coal store, boiler house for drying, store, laundry, &c. A powerful steam disinfector by Manlove, Alliot and Co., of Nottingham, has been erected, tested, and found to work well. The sanitary arrangements and the entire drainage system are most complete, and every possible precaution has been taken to secure immunity from smells, and preserve a healthy atmosphere both outside and inside.

### Brixham Cottage Hospital.

This new hospital is built on the Higher Brixham side of the town from designs by Mr. W. F. Tollit, of Totnes. It has three floors. On the ground floor are the wards, one for males and another for females, to the right and left of the matron's room, from which a view can be obtained of both wards. Adjoining the wards and overlooking the town is a balcony on which convalescent patients can enjoy the fresh air. There are also children's ward, patients' rooms, and nurses' rooms on the ground floor, whilst above are sitting and bed-rooms for three assistant nurses; sculleries, larders, &c., are below, and a lift is added for communication with the lower floor. Hot water is laid on throughout, on the upper floors being baths and lavatories. The building is of limestone with brick dressings. Messrs. Hazlewood Brothers are the builders, and the cost of the building is £2,000 in addition to furnishing.

### New Wing to West Ham Hospital.

Mr. J. Passmore Edwards has just opened a new wing of West Ham Hospital, the cost of which he is himself defraying. The present addition raises the number of beds by twenty-four, accommodation for sixty-three patients now being provided. The lower ward has been named after Mrs. Edwards. The new wing has cost £3,750.

### Bridgend Cottage Hospital.

The laying the foundation-stone of the Cottage Hospital in course of erection at Bridgend has just been performed. The building, which is designed to accommodate eight patients, comprises a ward for each sex, an isolation room, operating room, the usual domestic offices, and room for attendants and nurse. The contract has been let to Mr. Edward Preece, of Bridgend, at £1,500, and the architects are Messrs. Lambert and Rees of the same town.

